# Targeting myeloid-derived suppressor cells with gemcitabine to enhance efficacy of adoptive cell therapy in bladder cancer

**DOI:** 10.3389/fimmu.2023.1275375

**Published:** 2023-10-12

**Authors:** Sarah Bazargan, Brittany Bunch, Awino Maureiq E. Ojwang‘, Jamie Blauvelt, Annick Landin, Johannes Ali, Dominique Abrahams, Cheryl Cox, Amy M. Hall, Matthew S. Beatty, Michael Poch, Katarzyna A. Rejniak, Shari Pilon-Thomas

**Affiliations:** ^1^ Department of Immunology, Moffitt Cancer Center, Tampa, FL, United States; ^2^ Department of Integrated Mathematical Oncology, Moffitt Cancer Center, Tampa, FL, United States; ^3^ Comparative Medicine, University of South Florida, Tampa, FL, United States; ^4^ Cell Therapy Facility, Moffitt Cancer Center, Tampa, FL, United States; ^5^ Department of Genitourinary Oncology, Moffitt Cancer Center, Tampa, FL, United States

**Keywords:** tumor infiltrating lymphocytes (TIL), adoptive cell therapy (ACT), non-muscle invasive bladder cancer (NMIBC), neoadjuvant, gemcitabine, lymphodepletion

## Abstract

**Background:**

New therapeutics in development for bladder cancer need to address the recalcitrant nature of the disease. Intravesical adoptive cell therapy (ACT) with tumor infiltrating lymphocytes (TIL) can potentially induce durable responses in bladder cancer while maximizing T cells at the tumor site. T cells infused into the bladder directly encounter immunosuppressive populations, such as myeloid derived suppressor cells (MDSCs), that can attenuate T cell responses. Intravesical instillation of gemcitabine can be used as a lymphodepleting agent to precondition the bladder microenvironment for infused T cell products.

**Methods:**

Urine samples from bladder cancer patients and healthy donors were analyzed by flow cytometry and cytometric bead array for immune profiling and cytokine quantification. MDSCs were isolated from the urine and cocultured with stimulated T cells to assess effects on proliferation. An orthotopic murine model of bladder cancer was established using the MB49-OVA cell line and immune profiling was performed. MDSCs from tumor-bearing mice were cocultured with OT-I splenocytes to assess T cell proliferation. Mice received intravesical instillation of gemcitabine and depletion of immune cells was measured via flow cytometry. Bladder tumor growth of mice treated with intravesical gemcitabine, OT-I transgenic T cells, or combination was monitored via ultrasound measurement.

**Results:**

In comparison to healthy donors, urine specimen from bladder cancer patients show high levels of MDSCs and cytokines associated with myeloid chemotaxis, T cell chemotaxis, and inflammation. T cells isolated from healthy donors were less proliferative when cocultured with MDSCs from the urine. Orthotopic murine bladder tumors also presented with high levels of MDSCs along with enrichment of cytokines found in the patient urine samples. MDSCs isolated from spleens of tumor-bearing mice exerted suppressive effects on the proliferation of OT-I T cells. Intravesical instillation of gemcitabine reduced overall immune cells, MDSCs, and T cells in orthotopic bladder tumors. Combination treatment with gemcitabine and OT-I T cells resulted in sustained anti-tumor responses in comparison to monotherapy treatments.

**Conclusion:**

MDSCs are enriched within the microenvironment of bladder tumors and are suppressive to T cells. Gemcitabine can be used to lymphodeplete bladder tumors and precondition the microenvironment for intravesical ACT.

## Introduction

Bladder cancer is the sixth most common cancer in the United States (1–3). Patients diagnosed with non-muscle invasive bladder cancer (NMIBC) are treated with a combination of transurethral resection of bladder tumor (TURBT) and intravesical instillation of chemotherapy and/or Bacillus Calmette–Guérin (BCG), depending on risk stratification (4, 5). On average, 50% of NMIBC patients will experience a recurrence and approximately 15-20% of patients will progress to muscle invasive bladder cancer (MIBC), where the prognosis is significantly worse (6–8). The high recurrence rates in bladder cancer highlights the clinical gaps in bladder cancer management and calls for new therapeutics that can induce durable responses. One therapy that can potentially produce such responses is adoptive cell therapy (ACT) with tumor infiltrating lymphocytes (TIL).

ACT with TIL is a cancer immunotherapy that involves the expansion and infusion of a patient’s endogenous, tumor-specific T cells to treat cancer (9, 10). ACT with TIL has shown to produce durable anti-tumor responses in patients with metastatic melanoma. Due to the high mutational burden of both malignancies, ACT with TIL may induce similar responses in patients with bladder cancer (11, 12). Feasibility studies of growing TIL from bladder tumors support that TIL can be expanded from bladder cancer patient samples (13, 14). Additionally, previous work in murine models show that intravesical ACT with TIL is an effective method of treating smaller bladder tumors (15). However, preconditioning the tumor microenvironment prior to ACT has yet to be investigated.

The microenvironment of tumors is highly suppressive to T cells. The accumulation of immune populations, such as myeloid derived suppressor cells (MDSCs), regulatory T cells (T regs), and tumor associated macrophages (TAMs) can induce direct and indirect means of T cell suppression and is correlated with more aggressive disease (16–19). These immunosuppressive populations are more likely to attenuate anti-tumor responses of T cells transferred locally compared to T cells transferred systemically. We predict that the depletion of these accumulated populations in bladder tumors is likely to augment anti-tumor responses of TIL. One potential method to deplete these cells is using gemcitabine. Gemcitabine is an antimetabolite chemotherapy that is used routinely to treat bladder cancer and can have lymphodepleting properties at low doses (20, 21). In this work, we show that gemcitabine can be used as a preconditioning agent to deplete these immunosuppressive populations. When gemcitabine pretreatment is used in mice with larger tumors that are resistant to ACT with OT-I transgenic T cells, the anti-tumor responses of T cells instilled into the bladder are enhanced.

## Materials and methods

### Clinical specimens

All samples were collected under an IRB-approved study (MCC18142 and MCC20106). Patient demographics of samples were blinded until after analysis was complete.

### Urine collection and analysis

Urine samples were centrifuged at 500 x g for 5 minutes and supernatants were frozen for < 2 years at -80C before cytokine analysis. Cell pellets were treated with Red Blood Cell (RBC) lysis buffer (Biolegend, 420301) for 5 minutes and neutralized with PBS. Urine samples were centrifuged again at 500 x g for 5 minutes and passed through a 100 µm filter to remove cell clumps. An additional 70 µm filter was used if cell clumping was still present. Cells were resuspended in PBS and prepared for flow staining.

### Human tumor processing

Tumors were processed in a petri dish with human tumor digest buffer (human enzyme media), described previously (14). Tumors were mechanically digested using scalpels and the GentleMACS dissociator (Miltenyi). The digest was incubated with human enzyme media for one hour at 37° C. Cells were strained using a mesh cup and a 100 µm filter and subsequently treated with RBC lysis buffer.

### PBMC processing

Blood from healthy donors and bladder cancer patients were prepped by pooling sample tubes and diluting 2X with PBS. SepMate™ Tubes (STEMCELL Technologies, 85460) were used to isolate PBMCs by pipetting Ficoll into the bottom of the tube and layering with blood. The tubes were centrifuged at 290 x g for 10 minutes. The PBMC layer was isolated and centrifuged at 500 x g for 5 minutes. Cells were treated with RBC lysis buffer, as described above.

### Animals

Female C57BL/6 mice from Charles River were used for all *in vivo* experiments. Female OT-I transgenic mice (C57BL/6-Tg (TcraTcrb) 1100Mjb/J) from Jackson Laboratory were used for all experiments in which OT-I transgenic T cells or splenocytes were needed. All animals were housed at the Comparative Medicine Facility at the University of South Florida and humanely euthanized with CO_2_ inhalation followed by cervical dislocation when orthotopic tumors reached 250-350 mm^3^, subcutaneous tumors reached 1 cm in diameter, or when animals were in poor health.

### Cell lines and cell culture

We received MB49 bladder tumor cell line as a kind gift from Dr. Jeffery Schlom (National Cancer Institute, Bethesda, MA) (22). An ovalbumin (OVA) expressing fluorescent MB49 cell line (MB49OVA) was generated as described (15). Cells were cultured in complete media (CM), as previously described (15). Cells were passaged at 80% confluency, tested routinely for mycoplasma, and passaged no more than 10 times for experiments.

### Murine tumor models

For subcutaneous models, mice received subcutaneous injections of 5×10^5^ MB49-OVA cells. For orthotopic models, mice were anesthetized with isoflurane and kept warm using a heating pad. Gentle pressure was placed on bladders to evacuate any residual urine. Mice were then catheterized with 24G x 0.75 in catheters (Terumo) and pre-treated orthotopically with poly-L-lysine (Sigma) in PBS (50 µL at 1 µg/mL) for 10 minutes. Gentle pressure was used to empty bladders. Bladders were washed with 50 µL PBS and mice received intravesical instillation of 1×10^5^ MB49-OVA murine bladder cancer cells in PBS for 30 minutes. After the incubation period, catheters were removed, and mice were placed in recovery cages and monitored.

### Ultrasound

Six to ten days after tumor cell instillation, bladder tumors were monitored via the Vevo 2100 ultrasound system (FUJIFILM VisualSonics Inc.). Tumor measurements were recorded 1-2 times per week and tumor volumes were analyzed and calculated via the Vevo LAB ultrasound software (FUJIFILM VisualSonics Inc.).

### Murine tissue processing

After harvest, tumors were processed as previously described (15). Spleens were mechanically digested through a 100 µm filter using a syringe plunger and treated with RBC lysis buffer, described previously. Tumors used for initial immune profiling experiments were harvested at murine endpoint.

### Flow cytometry staining

Human urine cells, tumor digest, and PBMCs were stained with Zombie NIR (Biolegend, 423106) (1:1000 dilution) for live/dead staining. Cells were incubated in the dark at room temperature for 30 minutes and later washed with flow buffer (1.0 L DPBS/Modified without Calcium & Magnesium (HyClone, Cat No. SH30028.03), 5% Fetal Bovine Serum (FBS),1mM EDTA, 0.1% Sodium Azide). Fc block (Miltenyi, 130-059-901) was administered to each sample (1:100) and incubated in the dark at 4 C for 10 minutes. Samples were stained with the following antibodies for 20 minutes at 4 C: HLA-DR (BD, 555811), CD3 (Invitrogen, 17-0036-42), CD19 (Biolegend, 302212), CD56 (BD, 555518), CD14 (BD, 550787), CD11b, CD33 (Biolegend, 366618), LOX1 (Biolegend, 358604), and CD15 (Biolegend, 323040). Cells were washed with flow buffer and fixed with 2% paraformaldehyde (PFA) for 15 minutes at 4° C. Murine tumor digest was stained similarly to the above using murine Fc block (Tonbo, 70-0161-M001) and murine antibodies. Samples were stained with two panels: the first panel staining for CD4 (Biolegend, 116012), CD8 (BD, 553035), Nk1.1 (BioLegend, 108714), OVA-specific T cell receptors (MBL international, TB-5001-1), and CD45 (BioLegend, 103140) and the second panel staining for Ly6C (BioLegend, 128014), MHCII (BioLegend, 107636), CD45 (BioLegend, 103140), F4/80 (BioLegend, 123141), CD11b (BioLegend, 101228), CD19 (BD, 557399), CD103 (BioLegend, 121426), Ly6G (Tonbo, 20-1276-U100), and CD11c (Biolegend, 117339). For comparison of immune populations across murine treatment groups antibodies against CD45, CD4, CD8, Gr-1, and CD11b were used. All samples were run on FACSCelesta (BD) flow cytometer and analyzed using FlowJo V10 software.

### Cytokine analysis

All samples used for cytokine analysis were stored at -80C and analyzed < 2 years from sample collection. Human urine supernatants from bladder cancer patients and healthy donors were analyzed via the LEGENDplex HU Essential Immune Response Panel (Biolegend, 740929). The 13-plex was used to identify sample concentrations of IL-4, IL-2, CXCL10, IL-1b, TNF-a, CCL2, IL-17a, IL-6, IL-10, IFN-g, IL-12p70, and TGF-b. Murine urine and bladder fragment supernatants were analyzed via the LEGENDplex MU Cytokine Release Syndrome Panel (Biolegend, 741023). The 13-plex was used to identify sample concentrations of IFN-g, IL-10, CCL4, IFN-a, CXCL9, CXCL10, TNF-a, IL-6, VEGF, IL-4, CCL3, CCL2, and GM-CSF. All assays were performed according to manufacturer’s protocol and data analysis was performed using The LEGENDplex Data Analysis Software Suite (Biolegend).

### MDSC suppressor assay

Human MDSCs were isolated using CD15 MicroBeads (Miltenyi, 130-046-601). T cells were isolated from healthy donor PBMCs using an EasySep T cell isolation kit (STEMCELL, 17951) according to the manufacturer’s protocol. T cells were labeled with 5 µM CellTrace Violet (CTV) (Invitrogen, C34557) and incubated for 20 min, at 37° C, in the dark. T cells were stimulated with OKT-3 (10 µg/ml) and plated at decreasing ratios of MDSC: T cells. Cells were collected 72 hours later and analyzed by flow cytometry for CTV labeled T cells. Murine MDSCs were obtained from spleens of mice bearing subcutaneous MB49-OVA tumors. MDSCs were isolated from splenocytes using the MDSC Isolation Kit (Miltenyi, 130-094-538) according to manufacturer’s protocol. Splenocytes were obtained from OT-I TCR transgenic mice and labeled with CTV, as described above. T cells were stimulated with OVA-SIINFEKL peptide (1 µg/mL) and cocultured with MDSCs in a decreasing ratio of MDSCs to splenocytes. Cocultures were incubated for 66-72 hours and analyzed via flow cytometry for CTV labeled cells.

### Thymidine incorporation assay

Human T cells and MDSCs isolated previously were cocultured in decreasing ratios of MDSCs to T cells. Approximately 48 hours after plating, 1µCi of ^3^H thymidine in TIL CM was spiked into each well and left to incubate overnight. Cells were washed and harvested onto a filter mat and left to dry in an oven at 60° C. The dried filter mat was placed into a plastic sleeve, saturated with betaplate scint (PerkinElmer) and read using the MicroBeta Microplate Counter (PerkinElmer).

### IHC map of MDSCs

A portion of the resected tumors were cut into 4μm sections and stained for H&E, Ly6G, and CD31. These serial slide sections were scanned with a Leica Aperio AT2 digital Pathology Slide Scanner (Leica Biosystems, Vista, CA) with a 20x/0.7NA objective lens. The Tissuealign tool from Visiopharm version 2022.02 (Visiopharm A/S, Denmark) was used to co-register the whole slide SVS image files for the 3 IHC biomarkers with the H&E image. The H&E image was used to segment tumor and non-tumor regions under the guidance of the study pathologist. Next, a cell detection algorithm was used for each biomarker based on the pattern of DAB and Hematoxylin stains to label each cell as biomarker positive or negative with the thresholds set to be consistent for all samples. Finally, the intensity and spatial coordinates for each cell and vessel were reported. These were used for graphical reconstruction of the IHC maps for MDSCs, using the MATLAB software.

### Profiling immune populations and cytokines post gemcitabine treatment

To evaluate the effect of gemcitabine on immune cells in bladder tumors, mice with orthotopic MB49-OVA tumors (>50 mm^3^) were randomized and treated with intravesical instillation of gemcitabine (500 µg). Four days after instillation of therapy, bladders were harvested and processed as described above. Bladder digest was stained for overall immune cells, lymphocytes, and MDSCs. Samples were analyzed by flow cytometry and results were normalized to cell count and bladder mass. Using the same experimental design above, murine tumor-bearing bladders were cut into equal size fragments and plated in 1 mL of CM overnight. The supernatants were collected and analyzed for cytokines using the LEGENDplex kit previously described. In the untreated group, urine was also obtained by catheterizing mice and pulling out the liquid with a syringe. Urine samples were analyzed using the same LEGENDplex kit as the tumor supernatants.

### Treatment models

Mice received intravesical instillation of 1×10^5^ MB49-OVA cells for 30 minutes to establish orthotopic bladder tumors. Bladder tumor growth was measured via ultrasound and mice were separated into untreated, gemcitabine only (GEM), OT-I only (OT-I), and combination (GEM + OT-I) groups. Ten days after instillation of tumor cells, the GEM and GEM+OT-I groups received intravesical instillation of gemcitabine (500 µg in 50 µL PBS) for 30 minutes. Fourteen days after instillation of tumor cells, the OT-I and GEM+OT-I groups received intravesical instillation of 5×10^6^ OT-I transgenic T cells for a 3-hour incubation period. OT-I T cells were obtained by running splenocytes through T cell isolation columns (R&D Systems). Tumor volumes were recorded two times per week until endpoint of study.

### Statistical analysis

For comparisons between human specimens and *in vitro* experimental groups, a two-tailed Student’s *t* test was used. For *in vivo* experimental groups, a one-tailed Student’s *t* test was used. For experimental tumor growth curves, a Compare Groups of Growth Curves (CGGC) test was used. Outliers were identified using the ROUT method with a Q value of 0.1%. All statistical analyses were performed using GraphPad Prism and all *P* values ≤ 0.05 were determined to be statistically significant.

## Results

### MDSCs are enriched within the urine of bladder cancer patients and are suppressive to T cells

Previous reports have highlighted the importance of MDSCs in the pathogenesis and progression of bladder cancer (18, 23, 24). Therefore, we decided to focus on the impact of this specific population on T cells. We first wanted to assess whether there is an accumulation of MDSCs within the bladder microenvironment of cancer patients. Urine samples from healthy donors and bladder cancer patients were analyzed via flow cytometry. Demographics data regarding patient-derived samples are listed in **Table 1**.

**Table 1:** Patient sample demographics table Demographics data of patient-derived tissue samples.

Characteristic	n (%)
**Patients**	26
Sex	
Male	23 (88.5)
Female	3 (11.5)
**Age (mean)**	68.23
Grade	
High	24 (92.3)
Low	2 (7.7)

**Figure 1:**
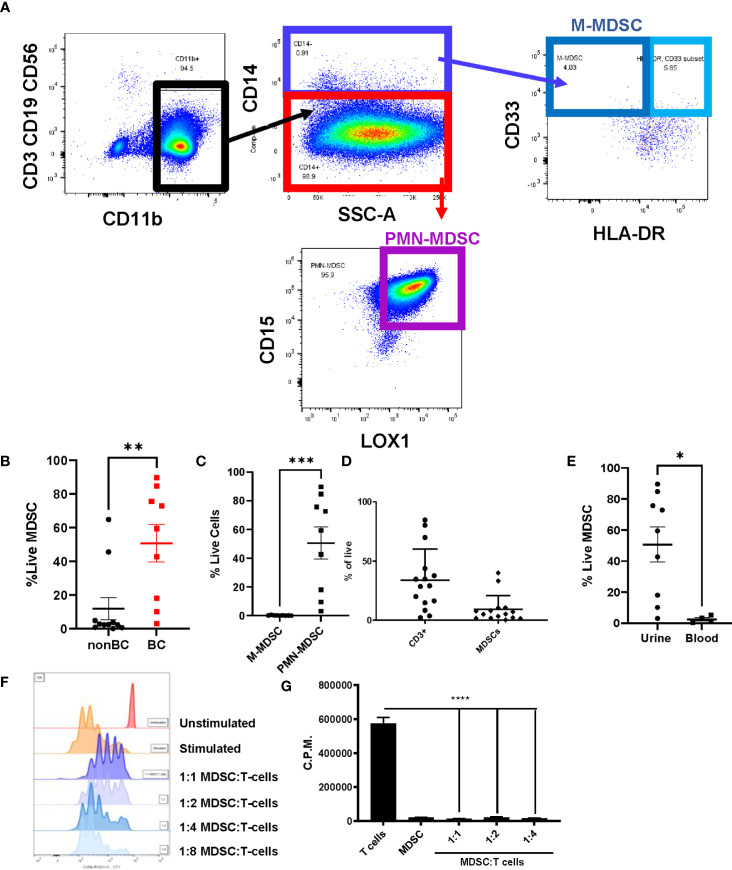
Urine of bladder cancer patients is enriched for MDSCs. **(A)** Gating strategy for MDSCs using urine cells from bladder cancer patient **(B)** Graph showing the percentage of live MDSCs in the urine of bladder cancer patients and healthy donors. n=9-11 per group. **(C)** Comparison of percentages of live PMN-MDSC and M-MDSC in the urine of bladder cancer patients, n=9. Composition of **(D)** immune cells in bladder tumors (n=15) and **(E)** MDSCs in urine and blood (n=4-9). **(F)** CTV flow cytometry analysis of 1:1, 1:2, 1:4, 1:8 coculture ratios of urinary MDSCs to T cells from healthy donor PBMCs. **(G)** Measurement of incorporation of tritiated thymidine in 1:1, 1:2, and 1:4 coculture ratios of MDSCs to T cells. All statistical analysis performed using two-tailed Student’s *t* tests. *, p ≤ 0.05; **, p ≤ 0.01; ***, p ≤ 0.001; ****, p ≤ 0.0001. With *P* values < 0.05.

Polymorphonuclear MDSCs (PMN-MDSCs) were identified as CD3^-^CD19^-^CD56^-^CD14^-^LOX1^+^CD15^+^ populations and monocytic MDSCs (M-MDSCs) were identified as CD3^-^CD19^-^CD56^-^CD14^+^CD33^+^HLA-DR^-^ populations (25) (**Figure 1A**). The sum of these populations equals the total amount of MDSCs in the sample. In comparison to the healthy cohort, the urine of bladder cancer patients was enriched for MDSCs (**Figure 1B**), with PMN-MDSCs making up most of these cells (**Figure 1C**). Interestingly, the levels of MDSCs within the bladder tumors were not as high as in the urine, potentially indicating that MDSCs may be shed from the tumor and released into the urine (**Figure 1D**). To determine whether this enrichment was specific to the bladder microenvironment or systemic, we obtained blood samples from patients and isolated the peripheral blood mononuclear cells (PBMCs). In comparison to the urine, PBMCs from bladder cancer patients contained very few MDSCs (**Figure 1E**).

**Figure 2:**
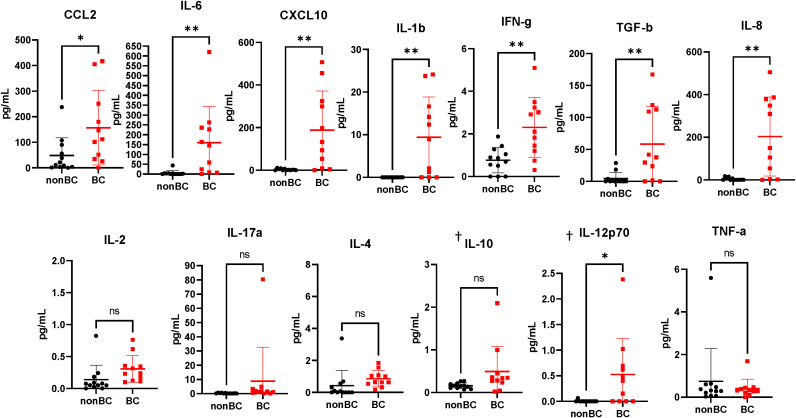
Urine supernatants from bladder cancer patients are highly enriched for cytokines. Cytometric bead array analysis of urine supernatants from bladder cancer patients and healthy donors. n=11-12 per group. Graphs marked with † are outside the limits of detection of the assay. All statistical analysis performed using two-tailed Student’s *t* tests. ns, non-significant; p > 0.05; *, p ≤ 0.05; **, p ≤ 0.01. With *P* values < 0.05.

To interrogate whether the accumulated MDSCs in bladder cancer patients are suppressive to T cells, coculture assays were performed using MDSCs isolated from the urine of patients and T cells isolated from healthy donor PBMCs. After stimulation of T cells with OKT-3, T cell proliferation was assessed through dilution of Cell Trace Violet (CTV). Coculture conditions with higher ratios of MDSCs resulted in less proliferation of T cells in comparison to cultures with fewer MDSCs (**Figure 1F**). Similar results were obtained by performing the same cocultures and measuring the incorporation of tritiated thymidine to assess T cell proliferation (**Figure 1G**). After determining that MDSCs accumulate in the bladder microenvironment and are suppressive to T cells, we next wanted to profile cytokine levels to better understand the microenvironment encountered by adoptively transferred T cells. Urinary cytokines have previously been used as a surrogate for immune activity in bladder cancer (26, 27). In comparison to healthy donors, CCL2, IL-6, CXCL10, IL-1b, IFN-g, TGF-b, and IL-8 were enriched within the urine of bladder cancer patients (**Figure 2**). CCL2 is associated with migration of monocytes and can serve as a chemoattractant for immunosuppressive cells (28, 29). IL-8 is associated with migration of neutrophils and has been associated with tumor progression in bladder cancer (30, 31). Enrichment of both cytokines may be supplementing further migration of MDSCs into the bladder microenvironment. IL-6 and IL-1b are proinflammatory cytokines that have been linked to bladder cancer progression (32, 33). CXCL10 promotes the migration of CXCR3 expressing cells, such as T cells, monocytes, NK cells, and tumor cells (34). The enrichment of CXCL10 is of particular interest due to its association with T cell infiltration into solid tumors (35–37). We have previously shown that CXCR3 signaling drives T cell infiltration into murine bladder tumors after intravesical ACT (15). We predict that these high CXCL10 levels are beneficial to intravesical ACT with TIL and warrants further study. Altogether, this data suggests that the urine of bladder cancer patients is enriched for MDSCs that are suppressive to T cells and that the microenvironment contains elevated levels of cytokines associated with inflammation, myeloid chemotaxis, and T cell chemotaxis.

### MDSCs are enriched within MB49-OVA tumors and are suppressive to T cells

**Figure 3:**
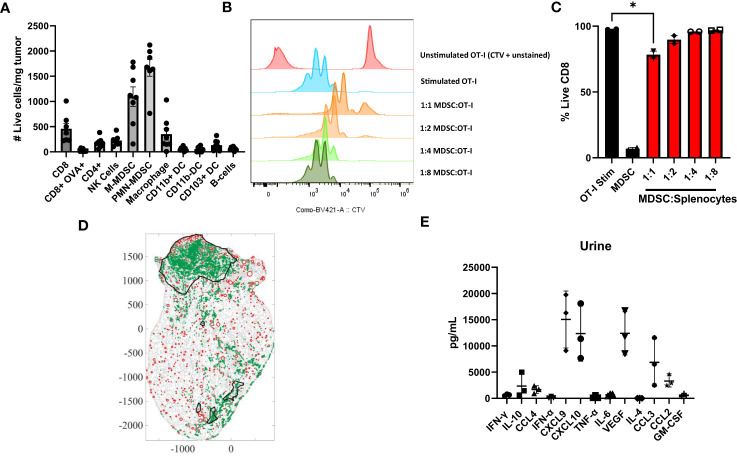
Murine bladder tumors are enriched for MDSCs that are suppressive to T cells. **(A)** Profile of immune populations in orthotopic MB49-OVA tumors normalized to number of live cells per milligram of tumor, n=7-8. **(B)** CTV flow cytometry analysis of 1:1, 1:2, 1:4, 1:8 coculture ratios of splenic MDSCs to OT-I T cells. **(C)** Graph showing expansion of CD8^+^ T cells in same coculture conditions. **(D)** Scanned tumor-bearing bladder tissue slides from mouse treated with intravesical instillation of OT-I T cells. Tumors are outlined in black, MDSCs are represented as green dots (identified via Ly6G staining), and blood vessels are represented as red circles (identified via CD31 staining). **(E)** Cytometric bead array analysis of urine supernatants from mice bearing orthotopic MB49-OVA tumors, n=3. All statistical analysis performed using two-tailed Student’s *t* tests. *, p ≤ 0.05. With *P* values < 0.05.

In our previous manuscript, we have shown that ACT with OT-I transgenic T cells is sufficient to control small MB49-OVA bladder tumors (15). In this work, we have adjusted our treatment schedules to use a tumor model that represents a larger tumor burden and fails to respond to ACT with OT-I T cells. We first wanted to validate that our murine bladder cancer model recapitulates the key characteristics of the human tumors highlighted in **Figure 1**. We profiled the immune microenvironment of orthotopic bladder tumors to assess relative MDSC enrichment. Briefly, orthotopic tumors were established through intravesical instillation of MB49-OVA murine bladder cancer cells. Bladder tumor growth was monitored via ultrasound measurement and bladders were harvested and processed and analyzed via flow cytometry to profile the immune infiltrate (**Figure 3A**). MDSCs were found to make up most of the immune cells in the tumor microenvironment and were about four times as prevalent as T cells. PMN-MDSCs made up the majority of the MDSCs; however, M-MDSCs seem to be more prevalent in the murine model in comparison to the human samples.

To determine whether murine MDSCs are suppressive to T cells, MDSCs isolated from spleens of mice bearing subcutaneous tumors were cocultured with OT-I splenocytes. T cells were stimulated with cognate ovalbumin antigen and proliferation was assessed through dilution of CTV. Matching the human data, murine T cells cocultured with higher ratios of MDSCs were less proliferative (**Figure 3B**). There was also an overall reduction in CD8^+^ T cells in coculture conditions where numbers of MDSCs matched the number of splenocytes (**Figure 3C**). Although we see a significant reduction in T cell proliferation at the 1:1 coculture condition. The most accurate representation within the bladder microenvrionment would be a 4:1 ratio of MDSCs to T cells. To show accumulation of MDSCs post-intravesical ACT, bladders of treated mice were fixed, stained, and digitized to visualize MDSC localization. A representative image is shown (**Figure 3D**). Mice that have received intravesical administration of OT-I T cells show a significant accumulation of MDSCs within their tumor (2986.2/mm^2^). The accumulation of these cells may contribute to immunosuppression within the tumor microenvironment, thus providing further rationale for incorporating a preconditioning regimen.

We next wanted to examine whether the cytokines in murine bladder tumors were similarly enriched to the cytokines in urine samples from the bladder cancer patients. Bladder fragments from mice bearing orthotopic tumors were plated and cultured overnight in media. Supernatants were collected the following day for analysis. Urine from bladder tumor bearing mice was also collected. Cytokine levels were assessed by comparing relative abundance across the same sample types. Within the urine samples, the relative abundance of CXCL9, CXCL10, VEGF, CCL3, and CCL2 was higher in comparison to the other cytokines tested (**Figure 3E**). Similarly, in the supernatant, the relative abundance of CXCL10, IL-6, and CCL2 was higher in comparison to the other cytokines tested (not shown). From the human data, the urine of bladder cancer patients had significantly upregulated levels of CCL2, IL-6, CXCL10, IL-1b, IFN-g, TGF-b, and IL-8 in comparison to the healthy donors. Therefore, the overexpression of CCL2, CXCL10, and IL-6 is shared in both murine and human samples. Mice do not have the IL-8 gene, and therefore we cannot make comparisons with the human data (38). Taken together, bladder tumors in our murine model have similar characteristics to the human samples through enrichment of MDSCs that are suppressive to T cells and an accumulation of cytokines that are associated with inflammation, myeloid chemotaxis, and T cell chemotaxis.

### Gemcitabine depletes immune cells in orthotopic bladder tumors

**Figure 4:**
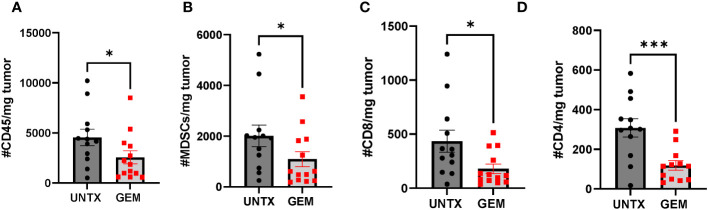
Intravesical instillation of gemcitabine reduces total number of immune cells. Graphs comparing total number of **(A)** CD45^+^ **(B)** MDSCs **(C)** CD8^+^ **(D)** CD4^+^ cells normalized to tumor mass in untreated mice and mice treated with intravesical instillation of 500 µg gemcitabine. n=12-13 per group. All statistical analysis performed using one-tailed Student’s *t* tests. *, p ≤ 0.05; ***, p ≤ 0.001. With *P* values < 0.05.

Gemcitabine is an antimetabolite chemotherapy that is already used in the clinic for the treatment of bladder cancer (39, 40). In addition to being tumoricidal, gemcitabine has been shown to have lymphodepleting properties at low concentrations and can deplete MDSCs (41). To assess the effects of gemcitabine on the immune microenvironment of murine bladder tumors, mice bearing orthotopic MB49-OVA tumors were treated with intravesical instillation of gemcitabine (500 µg/mouse). Four days later, bladder tumors were harvested and stained for overall immune cells (CD45^+^) (**Figure 4A**), MDSCs (**Figure 4B**), CD8^+^ T cells (**Figure 4C**) and CD4^+^ T cells (**Figure 4D**). In general, mice treated with intravesical instillation of gemcitabine had a significant reduction in overall immune cells, MDSCs, and T cells. Although gemcitabine is used to ablate MDSCs, the overall result is lymphodepletion as cell populations cannot be selectively depleted by the chemotherapy. However, the depletion of these bystander populations to make room for the infusion product may be an added benefit for T cell anti-tumor responses.

**Figure 5:**
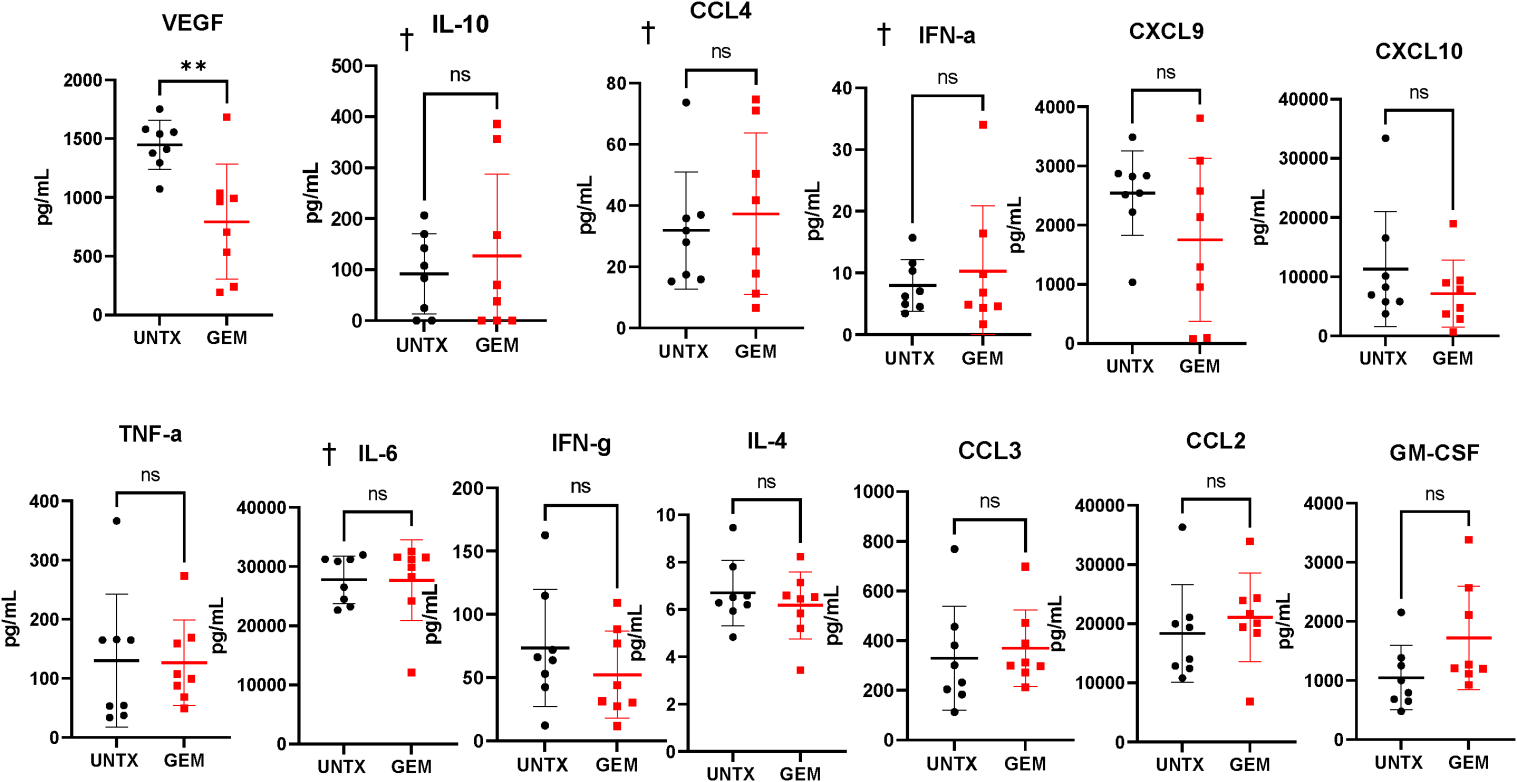
Gemcitabine treatment induces reduction of VEGF in tumor supernatants. Cytokine bead array analysis of supernatants collected from overnight cultures of tumor-bearing bladder fragments. n=8 per group. Graphs marked with † are outside the limits of detection of the assay. All statistical analysis performed using two-tailed Student’s *t* tests. ns, non-significant; p > 0.05; **, p ≤ 0.01. With *P* values < 0.05.

We observed that intravesical instillation of gemcitabine results in an overall lymphodepleting effect in the bladder microenvironment. We next wanted to determine whether this loss in immune cells results in an alteration in the cytokine levels in the bladder. Bladder tumor fragments of untreated mice and mice treated with intravesical gemcitabine were plated in media and the supernatants were collected 24 hours after plating for cytokine analysis. The only change in cytokine levels was a reduction in VEGF in the gemcitabine group (**Figure 5**). VEGF is important for tumor angiogenesis and promotes the expansion and migration of MDSCs (41, 42). There was a trend in an increase in GM-CSF in the gemcitabine group; however, this was not significant. While we see a reduction in immune cells in the bladder microenvironment, we do not see a reduction in the levels of cytokines important for T cell migration, namely CXCL9 and CXCL10. This may suggest that after treatment with gemcitabine, the availability of these cytokines do not change. Altogether, intravesical instillation of gemcitabine has a lymphodepleting effect on the bladder microenvironment with little impact to the availability of cytokines to T cells.

### Pretreatment of bladder tumors with gemcitabine enhances anti-tumor effects of ACT with tumor-specific T cells

**Figure 6:**
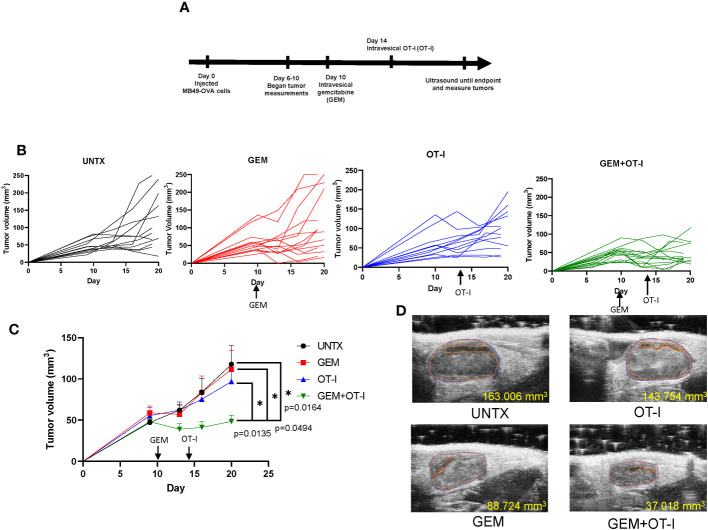
Pretreatment of gemcitabine combined with ACT improves anti-tumor responses. **(A)** Schematic showing timeline of treatments. **(B)** Individual growth curves of mice in the UNTX, GEM, OT-I, and GEM+OT-I groups. n=12-14 per group. **(C)** Average tumor growth curves for each treatment group. Statistical analysis performed via Compare Groups of Growth Curves (CGGC) test using 10,000 permutations. **(D)** Representative ultrasound images of each treatment group at day 20. *, p ≤ 0.05.

We have shown that intravesical instillation of gemcitabine has local lymphodepleting effects on the bladder tumor microenvironment. We predict that this pretreatment step will enhance the anti-tumor responses of adoptively transferred OT-I T cells into mice bearing MB49-OVA bladder tumors that are refractory to ACT. To test this, we established orthotopic MB49-OVA bladder tumors in mice and created four treatment groups: untreated (UNTX), gemcitabine treatment only (GEM), OT-I treatment only (OT-I), and combination gemcitabine and OT-I treatment (GEM+OT-I). Ten days after instillation of bladder tumor cells, mice in the GEM and GEM+OT-I groups received intravesical instillation of gemcitabine (500 µg). Four days after instillation of gemcitabine, mice in the OT-I and GEM+OT-I groups received intravesical instillation of OT-I T cells for a three-hour incubation period (5×10^6^ T cells). This represents a timepoint where ACT with OT-I T cells provides minimal anti-tumor responses due to the large size of the tumors. All mice underwent ultrasound two times per week and tumor growth was monitored until 20 days post tumor cell instillation (**Figure 6A**). Overall, monotherapies with either gemcitabine or OT-I T cells resulted in a small reduction in bladder tumor growth in comparison to untreated mice (**Figure 6B**). However, the combination of GEM and OT-I resulted in a significant reduction in tumor growth. By day 20, the monotherapy groups and the untreated group had similar tumor volumes while the combination group had a reduction in tumor volume (**Figures 6C, D**). Taken together, pretreating larger bladder tumors with gemcitabine before intravesical instillation of OT-I T cells results in enhanced anti-tumor responses in comparison to monotherapies of gemcitabine or ACT.

## Discussion

The tumor microenvironment is made up of several immune populations, many of which are suppressive to T cells (16). Specifically, MDSCs in bladder cancer have been associated with poorer clinical responses to BCG and neoadjuvant chemotherapy. Additionally, higher levels of MDSCs have been associated with higher grade malignancies in bladder cancer (23, 43). In this work, we have shown that MDSCs are enriched within the microenvironment of both human and murine bladder tumors. These MDSCs suppress activated T cells and exist in a microenvironment that is enriched for inflammatory cytokines, myeloid chemokines, and T cell chemokines. We show that intravesical instillation of gemcitabine can be used to locally lymphodeplete the bladder microenvironment with little impact to cytokines important for T cell migration. We predict that the high levels of CXCL10 can enhance migration of T cells into the bladder through interactions with CXCR3 (15). However, we do see a reduction in VEGF after treatment with gemcitabine, which warrants further investigation as to how this may impact ACT. We did expect to see changes in other myeloid cytokines and chemokines; however, it is possible that the kinetics of the other cytokines tested may require more time to show differences in the microenvironment. In our previous manuscript, we show that intravesical instillation of OT-I T cells is sufficient to control growth of bladder tumors. In this work, by design our bladder tumor burden was larger and ACT with OT-I alone was not able to control tumor growth. This was done intentionally to determine additive value of altering the tumor microenvironment. When gemcitabine is used to precondition the bladder microenvironment, we see an added anti-tumor benefit in combination with intravesical instillation of OT-I transgenic T cells. Even though our murine model had a larger tumor burden than our previous studies and with a human corollary of mixed stage disease, we still found an anti-tumor effect with combination intravesical therapy. This provides baseline data for the use of combination therapy for therapeutic pathway for smaller lower stage disease. Larger sample sizes correlating MDSCs to grade of tumor for smaller tumors would be a next step.

Cancer patients receiving T cell therapies typically undergo a systemic, nonmyeloablative preconditioning step with cyclophosphamide and fludarabine (44). This allows for the depletion of cytokine sinks and immunosuppressive populations that attenuate the activity of adoptively transferred T cells (45, 46). While a necessary preconditioning step, this lymphodepletion regimen is a significant source of patient toxicity and can lead to an overall reduction in quality of life (47, 48). Patients with bladder cancer who receive intravesical ACT would not require systemic chemotherapy due to local administration of the infusion product. Nonetheless, T cells adoptively transferred into the bladder are more likely to be affected by the immunosuppressive populations present in the bladder microenvironment. Here, we use gemcitabine to precondition the bladder microenvironment before administration of ACT. This allows for the local depletion of immunosuppressive populations that would attenuate T cell responses in the bladder without the toxicities associated with systemic chemotherapy.

One potential limitation in using gemcitabine as a lymphodepleting agent is separating out the tumoricidal effects from the lymphodepleting effects. One study using gemcitabine as a lymphodepleting agent showed that the tumoricidal properties of gemcitabine dominate when the tumors are small and the lymphodepleting properties dominate when the tumors are larger due to the accumulation of MDSCs and other immune populations (49). In this work, we allow tumors to grow larger (50 mm^3^) before we perform intravesical instillation of gemcitabine to optimize for lymphodepletion. Additionally, this is the tumor size where monotherapy with OT-I T cells has limited antitumor responses. Despite these limitations, gemcitabine is already used as a treatment for bladder cancer and its implementation as a preconditioning agent likely requires minimal adjustments to clinical practice (50). Another potential limitation of this work is the use of the OVA model for ACT. The immunogenicity of the OVA_257-264_ antigen expressed by the tumor cells may not be representative of the typical immunogenicity of tumor antigens in bladder cancer. Ideally, TIL from murine bladder tumors would be expanded *ex vivo* and used for ACT to more closely model the therapy administered in the clinic. Here, we use the OVA antigen specificity model to control for antigen recognition and reactivity of the infused T cell product.

We show that gemcitabine can be used as a preconditioning agent and that one round of instillation is sufficient to induce added benefits to ACT. Given that gemcitabine is administered locally to alter the bladder microenvironment, we expect the MDSCs to reconstitute back into the bladder over time. More work will need to be done to map out the optimal number of preconditioning cycles to enhance efficacy of ACT. Additionally, multiple infusions of T cells can be performed, allowing for the development of a highly personalized treatment regimen for each patient. Overall, using gemcitabine as a lymphodepleting agent can augment the anti-tumor responses of ACT.

## Data availability statement

The original contributions presented in the study are included in the article/supplementary material. Further inquiries can be directed to the corresponding author.

## Ethics statement

The studies involving humans were approved by Moffitt Scientific Research Committee and Advarra IRB. The studies were conducted in accordance with the local legislation and institutional requirements. The human samples used in this study were acquired from a by- product of routine care or industry. Written informed consent was received from participants in accordance with national legislation and institutional requirements. The animal study was approved by Institutional Animal Care and Use Committee (IACUC). The study was conducted in accordance with the local legislation and institutional requirements.

## Author contributions

SB: Conceptualization, Data curation, Formal Analysis, Methodology, Project administration, Supervision, Writing – original draft. BB: Conceptualization, Data curation, Formal Analysis, Methodology, Project administration, Supervision, Writing – review & editing. AO: Data curation, Formal Analysis, Supervision, Writing – original draft. JB: Methodology, Project administration, Writing – review & editing. AL: Data curation, Writing – review & editing. JA: Data curation, Writing – review & editing. DA: Project administration, Writing – review & editing. CC: Data curation, Project administration, Writing – review & editing. AH: Project administration, Writing – review & editing. MB: Conceptualization, Formal Analysis, Supervision, Writing – review & editing. MP: Formal Analysis, Supervision, Writing – review & editing. KR: Formal Analysis, Funding acquisition, Project administration, Supervision, Writing – review & editing. SP-T: Conceptualization, Formal Analysis, Funding acquisition, Methodology, Project administration, Supervision, Writing – review & editing.
